# Content analysis of locum tenens recruitment emails for anesthesiologists

**DOI:** 10.1186/s12913-018-3758-6

**Published:** 2018-12-19

**Authors:** Matthew DiMeglio, William Furey, Krzysztof Laudanski

**Affiliations:** 1 Department of Anesthesiology, Leonard Davis Institute for HealthCare, University of Pennsylvania, Philadelphia, PA 19146 USA; 2 Philadelphia College of Osteopathic Medicine, Philadelphia, PA 19131 USA

**Keywords:** Locum tenens, Anesthesiology, Content analysis, Email, Recruitment

## Abstract

**Background:**

Locum tenens continues to be an increasingly utilized employment option among healthcare organizations to cope with short-term provider vacancies. There exist no studies that explore the job characteristics of such assignments. The purpose of this study was to characterize the clinical responsibilities and compensation of anesthesiology locum tenens positions through content analysis of recruitment emails. Through this data, anesthesiologists interested in locum tenens will be better equipped to evaluate the merit of potential opportunities.

**Methods:**

The study was conducted using a compiled database of unsolicited emails received by one of the authors. A total of 241 emails containing 794 assignments were included during the period of 1/09/17 to 1/26/18 (383 days in total). The information was extracted using a standardized template and was entered into a database. Additional validation of the content was done using a data mining tool.

**Results:**

Most of the job opportunities originated from five staffing agencies. A total of 37, 25, and 17% of the assignments were allocated to hospitals, ambulatory surgical centers, and trauma centers respectively. The mean caseload for the assignments was between 8.5 and 11.1 cases per day. The mean daily work shift was 9.1 hours, and the average duration of the assignment was one week. The most frequently requested cases included general (74%), orthopedics (54%), and OB/GYN (51%). However, information regarding training qualifications and licensing was not routinely provided. Only 13.1% of assignments specified a system of medical documentation with paper charting being the most common. The mean hourly rate for locum anesthesiologists in our sample was $186.19, significantly higher than the national average of $127.88. Around 28% of staffing agencies covered the licensing expenses of specialists while 23% covered the expense of travels and 20% covered accommodation costs.

**Conclusions:**

Descriptions for locum tenens positions follow common anesthesiology practices and feature superior compensation to national estimates. However, vital information is often omitted from recruitment emails, and practice settings are highly variable. Anesthesiologists are urged to fully investigate opportunities before accepting based on recruitment emails. Managers should require more details to be provided in job offers.

**Electronic supplementary material:**

The online version of this article (10.1186/s12913-018-3758-6) contains supplementary material, which is available to authorized users.

## Background

Locum tenens, physicians hired on a temporary basis to fill vacancies in full-time positions, has become an increasingly common career option among the physician workforce dating back to 1985 [1–5]. Several factors are believed to contribute to the use of locum tenens such as a significant physician shortage, a larger insured population due to the Affordable Care Act (ACA), and geographical imbalances in physician supply [6–8]. This is particularly true in the case of anesthesiologists due to the shortage of providers coupled with an increase in surgical demand for an aging population [5, 9, 10]. Locum tenens physicians have served as an effective option for hospitals and private practices to address physician shortages to limit loss in productivity [5, 7, 11]. While some studies have suggested inadequacy of patient care among locum physicians, a recent study found similar 30-day mortality rates in Medicare beneficiaries treated by locum general internists compared to non-locum physicians [12–15]. Among physicians, the belief is that the adoption of this career option will provide a more equitable work-life balance, especially to younger doctors [16]. Some other commonly cited reasons for pursuing a career in locum tenens include schedule flexibility, high pay rate, lack of involvement in organizational politics, and opportunities for travel [5]. Earning a secondary income and the potential to increase retirement resources were additional reasons that motivated physicians to pursue locum tenens assignments [2]. Some commonly cited drawbacks to locum tenens include the likelihood of practicing away from home, stress from the uncertainty of the assignments, lack of benefits, and burden of credentialing process [5, 17].

There is virtually no literature characterizing the locum positions in anesthesiology. Since physicians select locum opportunities based on chacharacteristics such as pay rate, length of assignment, and patient load, it is strange that this information is not publicly available [5]. A recent survey did not even mention locum tenens as a component of the anesthesiology workforce, even though a significant minority of anesthesiologists work on a part-time basis [10, 18]. Gathering any insight into how recruitments are done carries important data for potential applicants and hospital management. This is especially true in the field of anesthesiology, where the potential mismatch between a hospital's ability to provide surgery services, patient demand, and severity in interruption of anesthesiology services delivery may have a profound effect on hospital operations and revenue.

The purpose of this study was to provide the first analysis of the anesthesiology locum tenens market through content analysis of recruitment emails by staffing agencies. Content analysis of these emails should help to better characterize the locum anesthesiology market by extracting information such as assignment locations, practice characteristics, clinical duties, and compensation from recruitment emails. This data will provide anesthesiologists a benchmark for comparison of different locum tenens opportunities.

## Methods

### Sample

This is a cross-sectional, descriptive study that evaluated a database of emails received by one of the authors (KL). The emails included in this study were delivered after one of the authors registered in one of the one of the locum tenens staffing agency during the Society of Critical Care Medicine Congress in 2015. The collection of email was carried out after a delay period. After an eight-month lag period, a total of 521 emails were received during the period of 1/09/17 to 1/26/18 (383 days in total). There was no attempt to communicate with any of the soliciting companies.

### Inclusion criteria

The sample followed a set of inclusion criteria and these included: 1) emails regarding temporary anesthesia assignments 2) e-mails recruiting for temporary, short-term positions. Therefore, emails that delivered information regarding permanent positions, targeted recruitment of specialists other than anesthesiologists, and emails that included coding information were excluded from the content analysis in this study. After applying inclusion criteria, a total of 241 emails containing 794 assignments were included for further analysis (46.3% of total emails).

### Data collection

The content of all emails was coded using a database with a total of 82 variables. The content of the emails was analyzed by MD and WF using a standardized template. The variables, along with their definitions and coded values, is presented as Additional file 1. The intercoder reliability was derived from the average percent agreement among a sample of five assignments and amounted to 96.39% agreement among the coded variables.

Compensation was presented either as an hourly or per diem rate. If compensation was presented as a per diem rate but the daily work hours were not provided, the per diem rate was divided by the mean work hours of all assignments.

### Statistical analysis

The emails were analyzed using descriptive statistics. Median and interquartile range (IQR) of first and third quartile were reported for continuous data. Chi-Square testing was used to evaluate the degree of association among categorical variables. Comparison of compensation by the state was assessed using the Kruskal-Wallis test. Differences were deemed significant if the two-tailed statistic was below 0.05. Statistica 11.0 was used to process data (Tulsa, AZ).

## Results

### Characteristics of the sample

**Fig. 1:**
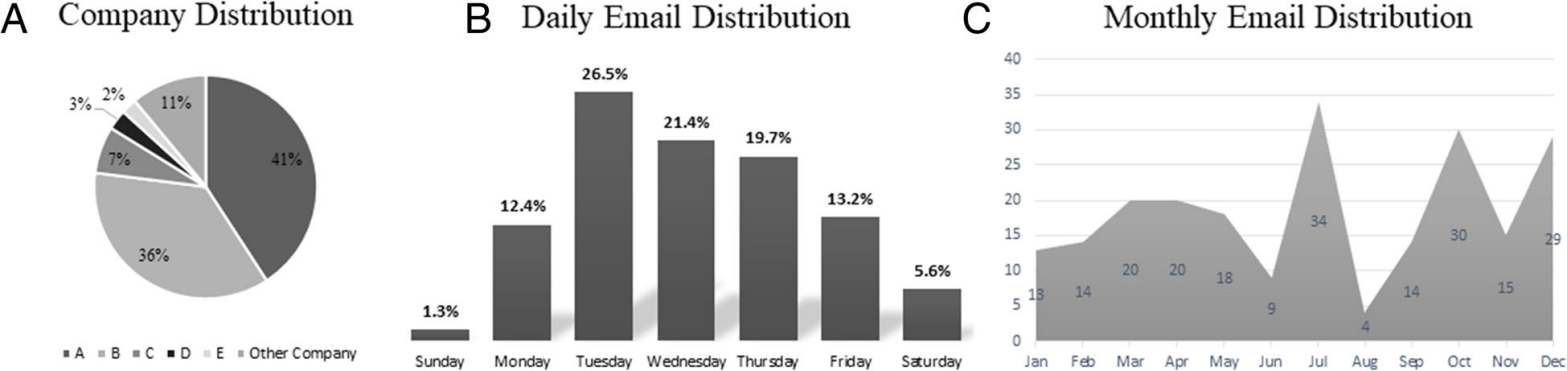
Most of the assignments were offered by five companies with 77% of all assignments coming from two companies (**a**). Tuesday was the most common day that emails were sent, and 6.9% of emails were received during the weekend (**b**). The highest influx of emails was observed in July while the fewest emails were received in August (**c**)

A total of 241 emails that contained 794 assignments were received during the period of 1/09/17 to 1/26/18 (383 days in total). Five staffing agencies generated most of the emails with Tuesday being the predominant day for the delivery of most emails (Fig. 1a & b). Interestingly, the seasonality of email influx was much less pronounced in the annual cycle (Fig. 1c). We did not spot an increase in an email around holidays or during the summer.

### Practice setting

**Fig. 2:**
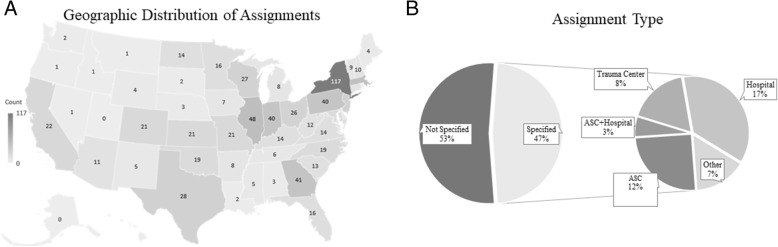
New York, Indiana, and Illinois offered the most assignments while Alaska, Rhode Island, and Utah did not offer any assignments (**a**). Interestingly, only 9.3% of the total assignments were within the Delaware Valley Tri-State area (NJ, PA, and DE). Of the 47% of assignments that specified a center type, the hospital setting was the most common (17%) followed by the ambulatory surgical center (12%) and trauma center (8%). The “other” section consisted of a variety of settings such as private practice, clinic, and government facility (**b**)

The geographical distribution of the assignments is presented in Fig. 2a. From our sample, significantly more job opportunities were available in New York (14.69%), followed by Illinois (6.05%) and Massachusetts (5.79%) (χ^2^ = 16.85; *p* < 0.001). Conversely, no assignments were available in Alaska, Rhode Island, and Utah. The hospital setting was the most common site for specialist recruitment (37%) followed by ambulatory surgical centers (25%), and trauma centers (17%) (Fig. 2b) (χ^2^ = 284; *p* < 0.001). A total of 42 and 28% of assignments were allocated to inpatient and outpatient settings respectively, while 30% of assignments were mixed (. Level 2 trauma centers had the greatest need accounting for 45% followed by 38% in level 1 trauma centers and 17% in level 3 trauma centers.

**Fig. 3:**
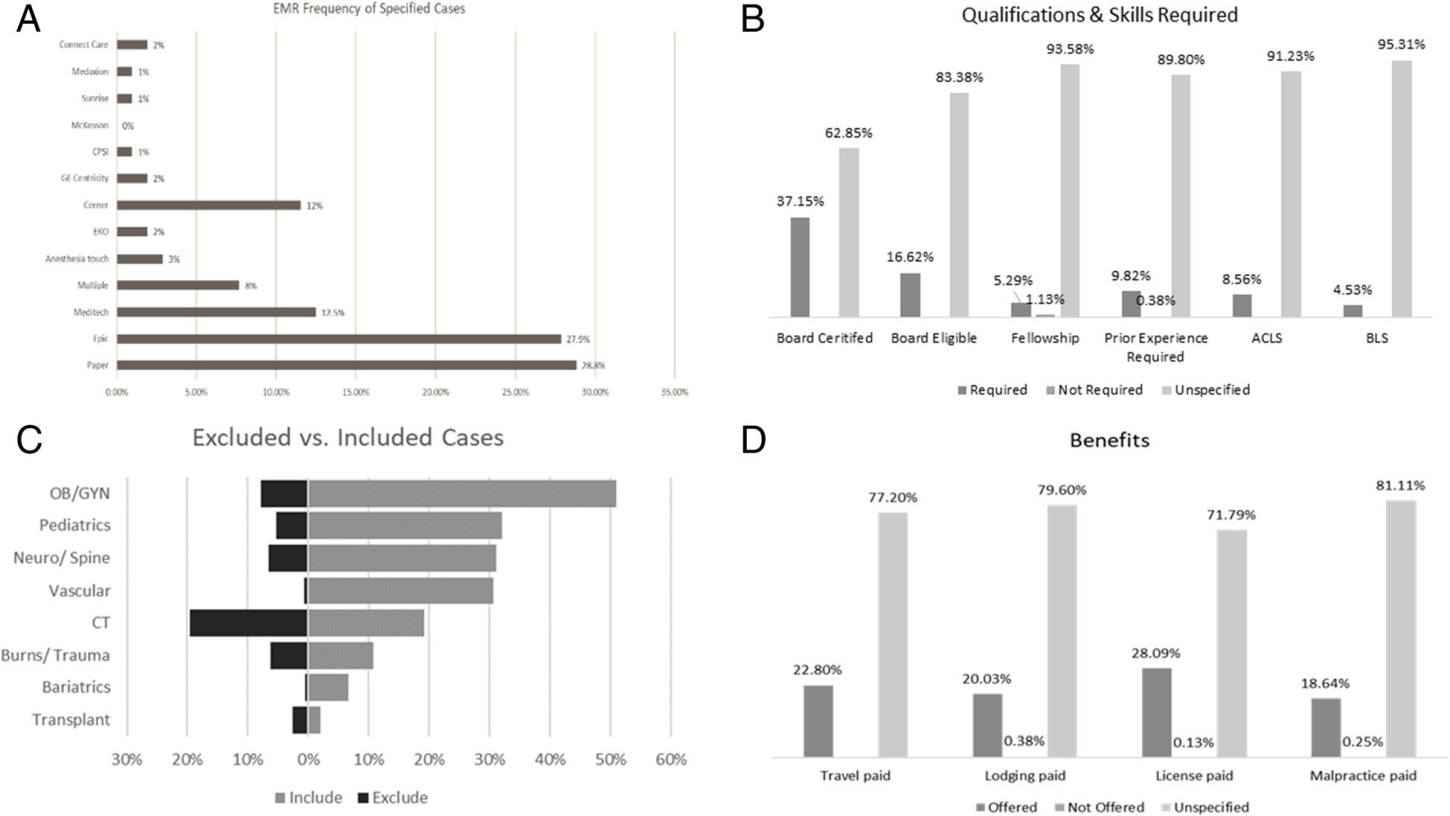
Paper charting was the most commonly cited form of medical documentation (28.8%) followed by Epic® (27.9%) and Meditech® (12.5%) (**a**). Board certification was the more common prerequisite specified than board eligible (37.15%) (**b**). The most commonly requested cases included general (74%), orthopedics (54%), and OB/GYN (51%) while cardiothoracic anesthesia was more commonly excluded (20%) that included (19%) (**c**). Prominent benefits specified included paid licensure (28.09%) and covered travel expenses (22.80%) (**d**)

Approximately 50% of email opportunities specified the role of the anesthesiologist with certified registered nurse anesthetist (CRNA) supervision far more common than being a direct provider (43% vs. 7% respectively). The median number of anesthesiologists and CRNAs working at the assignment was specified as 6 (IQR, 4–12) and 8 (IQR, 3–13) respectively, but this difference was non-significant. Of the 13.1% of assignments specifying a system of medical documentation, paper charting was the most common method (28.8%) followed closely by Epic® (27.9%) and Meditech® (12.5%) (Fig. 3a). Also, around 8% of the assignments required proficiency in multiple documentation software.

Most commonly cited length of the assignment was “ongoing” which accounted for 65.6%, while 23.8% of the emails specified a discrete time-frame. The average length of the assignment for the short-term assignments was 3.6 weeks with the most common length of the assignment being one week. Only 4.7% of assignments commented on the potential to transition to a permanent position. Finally, the usual lag between assignment and the time when email was received was 46.6 days.

### Requirements and description of clinical assignments

Only 48.4% of assignments specified explicitly whether a medical license was required for the position. The most common licensing requirement was to have an active license in the state of the assignment (42.7%) followed by temporary privileges (3.2%) and any state license (2.5%). Of the assignments specifying any state license, the majority were in Arizona, and 2.3% were government facilities. Prior experience was uniformly required by all agencies (96%). In most of the cases, being a board-certified anesthesiologist was an explicit requirement as well (Fig. 3b). In a few cases (5.3%), a fellowship was required mostly in the form of cardiac anesthesia (1.9%). Interestingly, only on a few occasions having fellowship correlated with the inclusion of the cardiac anesthesia. Most of the emails required prior experience to be considered for the position (Fig. 3b).

The median caseload for the assignment reported as minimum and maximum caseload by the agency was between 8 (IQR, 5–10) and 10 (IQR, 8–15) cases per day. This number represents data from approximately 10% of the reviewed assignments. Almost all emails (98.4%) specified at least one case type, and the median number of case types reported was 4 (IQR, 2–7). The most requested cases were general surgery (74%), orthopedics (54%), and OB/GYN (51%) procedures. The least requested cases were robotics (3%), transplant (2%), and geriatrics/palliative care (2%) procedures (Fig. 3b). The most frequently excluded cases were cardiothoracic (20%), OB/GYN (8%), and neurological (7%) procedures. Interestingly, cardiothoracic was the only case type that was more frequently excluded (20%) than included (19%) when specified for an assignment (Fig. 3c).

### Compensation and benefits

The median daily work hours were 8.5 (IQR,8–10). Among all assignments that provided compensation information (8.3% or *n* = 66), the mean hourly rate for locum anesthesiologists in our sample was $186.19 with a standard deviation of $30.72. There was statistically significant variation in the salary offered depending on the state of assignments (*p* = 0.0081) (GA = $2200 ± 400; NC = $1447 ± 392; NJ = $1630 ± 155; NY = $1760 ± 358; OK = $2025 ± 450; PA = 2120 ± 415). Salary did not differ between agencies (data not shown).

In terms of ancillary compensation packages such as paid travel, lodging, malpractice insurance, and licensing, whether these expenses were covered was not frequently specified by the staffing agency. Complimentary assistance with licensing costs was the most frequently cited (28.1%), followed by paid travel (22.8%) and lodging (20.03%) (Fig. 3d). Also, a very small percentage (3.5%) of assignments specified that these ancillary benefits were not covered by the staffing agency. While most of the compensation packages did not differ among agencies, there were significant differences in malpractice coverage between agencies (χ^2^ = 99.3; *p* < 0.001).

## Discussion

To our knowledge, this is the first study investigating the characteristics of locum tenens position in the field of anesthesiology outside of advertisement materials by staffing agencies. This study also analyzed the amount of information staffing agencies provide to locum tenens physicians regarding potential assignments. While this data provides insight into the aspects of a locum tenens assignment for anesthesiologists, most recruitment emails contained inadequate information for a physician to make an informed decision to accept or deny the opportunity. From that perspective, emails were a “recruitment” tool for future recruitment. There is no data what is the optimal amount of information encapsulated in the initial email to incentivize the physician to place a phone call to a staffing agency. This creates a potential for future research direction. Prior research suggests that locum physicians consistently consider factors such as pay rate, licensing, and the ability to travel when choosing among assignments [2, 5]. Stress from uncertainty surrounding the requirements of the assignment appears to be a significant drawback for locum tenens physicians [5]. Despite this information, staffing agencies failed to provide information that would have partially satisfied the concerns of locum tenens physicians. Our study found that only 8.3% of assignments provided payment information, 28.1% specified licensing assistance, and 22.8% specified travel reimbursement to the location. Only 10% of assignments specified the average caseload, 32% specified mean daily hours, and a very small minority reported the various requirements of the position such as prior experience or fellowship training.

However, the data collected offers an interesting insight into the practice setting of a locum tenens anesthesiologist. The most sought-after requirement by assignments was a prior experience, but no emphasis on the fellowship. Also, there was no spike in hiring around the holidays and periods with high staff turnover because of the training organization in the USA (June/July). Finally, hospitals were primary destinations for locum tenens. This suggests that locum tenens in anesthesiology are employed to close an existing gap and maintain the productivity of the requester which is consistent with prior studies of locum tenens in outside of anesthesia needs.

Another interesting discovery was the distribution of electronic medical records (EMR) systems in place across our sample. Although only 15% of job opportunities specified the health record system in place, the most frequently-cited modality was paper charting. While this may be a function of our limited sample size, it would be interesting to see if this feature is a purposeful strategy to recruit physicians that may be concerned with using an unfamiliar EMR. The prevalence of paper charting may also be reflective of the facilities seeking locum tenens coverage. One could speculate that smaller anesthesia groups would be less inclined to employ EMR, but the introduction of the ACA forced virtually all practices to adopt EMR. Alternatively, not all anesthesia locations are equipped with EHR, so both systems may co-exist side by side [19, 20].

One of the most interesting findings was an analysis of the wages provided. This value was higher than the 2017 Bureau of Labor Statistics mean hourly wage estimate of $127.88 for anesthesiologists, which is based off a mean annual wage of $265,990 and a “year-round, full-time” estimate of 2080 hours [21]. The calculated wage was also slightly higher than the $180.77 hourly rate calculated from a $376,000 mean annual salary in a recent review from a physician recruitment agency [22]. To standardize the rates, the 2080 hour “year-round, full-time” estimate from BLS was used to calculate the hourly rate reported by the physician recruitment agency. Considering that the most common length of the assignment was one week, the mean income from one assignment amounts to $6507.72 (7 days and 9 hours of work per day). Considering that most of these assignments can be contracted on the Internal Revenue Service (IRS) Form 1099, the financial incentive is quite significant and competitive to academic or private salaried positions. However, the financial benefit could not be measured since most assignments did not specify if related expenses such as licensing, travel, and lodging were reimbursed.The analysis if compensation and utilization of 1099 deductions would be extremely useful for staffing agencies and hospital managers to adjust the pay structure to make locum tenens assignment salary competitive enough to compete on the market without affecting bottom line to profoundly.

Analysis of job postings also offers additional information for hospital managers. The hospital setting is the primary source of demand for locum positions. In the US, the growth of the ambulatory surgical center (ASC) is remarkable but is not reflected in our study. This may suggest that hospitals need higher flexibility in their capacity to deliver anesthesia services. Historically, this was a case even before the introduction of the ACA [2, 14]. The length of the assignment was quite long suggesting that locum tenens are not hired to cover holidays, but they fill a sustained period of higher demand. Focus on surgical and orthopedics cases further suggest rapid growth in the service areas which is concomitant with national trends [23, 24]. The sustained demand may suggest that hospitals continue to copy with providers shortage. This suggests that pay rates should be increased to match those provided by locum tenens in the form on moonlighting opportunities or increase in salary [25]. Finally, the low percentage of trauma centers in our study reflect state legislation that causes the hospital to over-staff to provide anesthesia services even when the trauma service is idle. It is also interesting that compensation packages varied greatly among staffing agencies indicating high variability in the market. Consequently, managers should carefully study which companies provide the most attractive compensation packages to provide a reliable influx of providers. This is of particular concern considering that most of the locum tenens physician will operate on 1099 [2, 25]. This means that they can carry the expenses of running their business (being a locum tenens providers), but extra expenditure may significantly cut into their bottom line. Modest pay variance in our study sample suggests that offering different amenities to the income (license fees, travel, malpractice) may be the most effective way to attract the candidates. Again more research is needed considering that the data about the needs and expectation of providers, staffing agencies and requestors are virtually non-existent. For example, investigating the relationship between effective recruitment and offered compensation (and amenities with it) may reveal not straightforward regression considering that several motivators are non-monetary for locum tenens physicians as opposed to another form of employment [25].

Our study has some limitations. It is a descriptive study of a convenience sample of unsolicited email. Most of the assignment requests were from states neighboring the site of research, which suggests that the data may not be representative of the United States population. Although the study was exploratory in nature, the coders understood the purpose of the study suggesting a potential bias. Several factors were only able to be analyzed in terms of absolute frequency. Although we analyzed emails with at least 2 pieces of extractable information, some emails still contained rudimentary data.

Despite several limitations, some of the findings of the study are corroborated by other data. EPIC remains the most dominant Anesthesia Information Management Systems (AIMS) [26]. The emails that advertised “any license” offered only positions within government facilities which are in line with Veterans Affairs (VA) privilege system [27]. The locum tenens compensation variance followed national trends [18]. The self-employed anesthesiologist earns more as compared to employed ones [18]. The average time spent with the patient roughly matched nationwide trends [18]. Supervision of CRNA is commonplace [10]. These details confirm that although our email collection is a convenience sample, it is also a source of robust data considering the nature of the study.

## Conclusions

The increase in the number of requests for locum tenens coverage documented over the past few years portrays the importance of a closer examination of the qualitative and quantitative data apparent in locum tenens opportunities [18, 28]. When evaluating these opportunities, complete information is necessary to make guided decisions confidently and efficiently match opportunities to interested candidates. Standardization of this information including all pertinent information necessary therein may be an avenue for improvement for physician recruiting companies. From the data we did retrieve, our results confirm other findings that the hospital setting is currently the most frequent setting requiring the need for locum tenens coverage.

The paucity of research in this field suggests that more studies are required to enhance the medical community’s understanding of the efficacy, practicality, and economic utility of locum tenens coverage. With current projections indicating the demand for locum tenens is only set to increase ahead, more research is required to evaluate the logistics and costs of successfully credentialing and fulfilling an opening [5]. Locum tenens offers another avenue for keeping pace with healthcare demand in America. As the efficacy and employment process of these practitioners becomes clearer to the medical community, enhanced utilization of this resource should be realized in helping medical facilities meet their short-term obligations.

## Additional file

Additional file 1: The key for data coding. This is the key used for coding the data from the email into numerical format for the further analysis. (DOCX 23kb)

## Abbreviations

ACA: Affordable Care Act
AIMS: Anesthesia Information Management Systems
CRNA: Certified registered nurse anesthetist
IRS: Internal Revenue Service
VA: Veterans Affairs
