# Supplementary material for: Content analysis of locum tenens recruitment emails for anesthesiologists
Source: BMC Health Serv Res. 2018 Dec 19;18:981. doi: 10.1186/s12913-018-3758-6 (PMC6300003; doi:10.1186/s12913-018-3758-6)
Supplement: Supplementary file 1 — The key for data coding. This is the key used for coding the data from the email into numerical format for the further analysis. (DOCX 23kb) [file 12913_2018_3758_MOESM1_ESM.docx]

**Table S1: Coding Template**

| **Variable Number** | **Variable Description** | **Variable Type** | **Variable Values** |
| --- | --- | --- | --- |
| 1 | Date of Email | Continuous | MM/DD/YYYY |
| 2 | Time of Email | Continuous | Military Time |
| 3 | State of Assignment | Categorical | State Abbreviation |
| 4 | Type of Center | Categorical | x - Not specified 1 - Ambulatory Surgery Center (ASC) 2 - both ASC & Hospital 3 - Level II Trauma 4 - Teaching Facility 5- Hospital 6 - Intensive Care Unit (ICU) 7 - Private Practice 8- Level 1 Trauma 9 - Clinic / Outpatient Office 10- Hospital Owned Practice 11- Level III Trauma 12-Spine Center 13- Gov't facility 14- eICU / Telemedicine |
| 5 | Recoded Center Type | Categorical | x - Not specified 1 - Ambulatory Surgery Center (ASC) 2 - both ASC & Hospital 3 - Trauma (All Levels) 4 - Teaching Facility 5- Hospital 6 - Intensive Care Unit (ICU) 7 - Private Practice 9 - Clinic / Outpatient Office 10- Hospital Owned Practice 12-Spine Center 13- Gov't facility 14- eICU / Telemedicine |
| 6 | Type of Electronic Medical Record System | Categorical | x - Not specified A- Paper B-Epic C-Meditech D-Multiple E-Anesthesia Touch F - EKO G- Cerner H- GE Centricity I - CPSI J - McKesson K-Sorian L-Sunrise M - Medaxion N - ConnectCare O - E CLINICAL |
| 7 | Number of Beds at Facility | Continuous | Number |
| 8 | Minimum number of cases per physician | Continuous | Number |
| 9 | Maximum number of cases per physician | Continuous | Number |
| 10 | Number of Physicians at Facility | Continuous | Number |
| 11 | Number of CRNAs at Facility | Continuous | Number |
| 12 | Total Number of Providers at Facility | Continuous | Number |
| 13 | Inpatient vs. Outpatient Facility | Categorical | 1 - Inpatient 2 - Outpatient 3 - Both |
| 14 | "Bread and Butter" cases included | Categorical | 0 - Not specified 1 - Yes (Nothing Specified) 2- Yes (Experience Required) 3 - Yes (Fellowship Required) 4- Yes (Fellowship and Experience Required) |
| 15 | Bariatrics cases included | Categorical | 0 - Not specified 1 - Yes (Nothing Specified) 2- Yes (Experience Required) 3 - Yes (Fellowship Required) 4- Yes (Fellowship and Experience Required) |
| 16 | General cases included | Categorical | 0 - Not specified 1 - Yes (Nothing Specified) 2- Yes (Experience Required) 3 - Yes (Fellowship Required) 4- Yes (Fellowship and Experience Required) |
| 17 | Cardiothoracic cases included | Categorical | 0 - Not specified 1 - Yes (Nothing Specified) 2- Yes (Experience Required) 3 - Yes (Fellowship Required) 4- Yes (Fellowship and Experience Required) |
| 18 | Pulmonary/Critical Care cases included | Categorical | 0 - Not specified 1 - Yes (Nothing Specified) 2- Yes (Experience Required) 3 - Yes (Fellowship Required) 4- Yes (Fellowship and Experience Required) |
| 19 | "Neurosurgery/Spine cases included | Categorical | 0 - Not specified 1 - Yes (Nothing Specified) 2- Yes (Experience Required) 3 - Yes (Fellowship Required) 4- Yes (Fellowship and Experience Required) |
| 20 | Dental cases included | Categorical | 0 - Not specified 1 - Yes (Nothing Specified) 2- Yes (Experience Required) 3 - Yes (Fellowship Required) 4- Yes (Fellowship and Experience Required) |
| 21 | Burns/Trauma cases included | Categorical | 0 - Not specified 1 - Yes (Nothing Specified) 2- Yes (Experience Required) 3 - Yes (Fellowship Required) 4- Yes (Fellowship and Experience Required) |
| 22 | Transplantation cases included | Categorical | 0 - Not specified 1 - Yes (Nothing Specified) 2- Yes (Experience Required) 3 - Yes (Fellowship Required) 4- Yes (Fellowship and Experience Required) |
| 23 | Pediatric cases included | Categorical | 0 - Not specified 1 - Yes (Nothing Specified) 2- Yes (Experience Required) 3 - Yes (Fellowship Required) 4- Yes (Fellowship and Experience Required) |
| 24 | Podiatry cases included | Categorical | 0 - Not specified 1 - Yes (Nothing Specified) 2- Yes (Experience Required) 3 - Yes (Fellowship Required) 4- Yes (Fellowship and Experience Required) |
| 25 | Pain management cases included | Categorical | 0 - Not specified 1 - Yes (Nothing Specified) 2- Yes (Experience Required) 3 - Yes (Fellowship Required) 4- Yes (Fellowship and Experience Required) |
| 26 | Plastic surgery cases included | Categorical | 0 - Not specified 1 - Yes (Nothing Specified) 2- Yes (Experience Required) 3 - Yes (Fellowship Required) 4- Yes (Fellowship and Experience Required) |
| 27 | Palliative Care/Geriatric cases included | Categorical | 0 - Not specified 1 - Yes (Nothing Specified) 2- Yes (Experience Required) 3 - Yes (Fellowship Required) 4- Yes (Fellowship and Experience Required) |
| 28 | Regional anesthesia/Nerve blocks cases included | Categorical | 0 - Not specified 1 - Yes (Nothing Specified) 2- Yes (Experience Required) 3 - Yes (Fellowship Required) 4- Yes (Fellowship and Experience Required) |
| 29 | Robotic surgery cases included | Categorical | 0 - Not specified 1 - Yes (Nothing Specified) 2- Yes (Experience Required) 3 - Yes (Fellowship Required) 4- Yes (Fellowship and Experience Required) |
| 30 | Obstetrics/Gynecology cases included | Categorical | 0 - Not specified 1 - Yes (Nothing Specified) 2- Yes (Experience Required) 3 - Yes (Fellowship Required) 4- Yes (Fellowship and Experience Required) |
| 31 | Otorhinolaryngology cases included | Categorical | 0 - Not specified 1 - Yes (Nothing Specified) 2- Yes (Experience Required) 3 - Yes (Fellowship Required) 4- Yes (Fellowship and Experience Required) |
| 32 | Ophthalmology cases included | Categorical | 0 - Not specified 1 - Yes (Nothing Specified) 2- Yes (Experience Required) 3 - Yes (Fellowship Required) 4- Yes (Fellowship and Experience Required) |
| 33 | Endoscopy/Gastrointestinal cases included | Categorical | 0 - Not specified 1 - Yes (Nothing Specified) 2- Yes (Experience Required) 3 - Yes (Fellowship Required) 4- Yes (Fellowship and Experience Required) |
| 34 | Vascular cases included | Categorical | 0 - Not specified 1 - Yes (Nothing Specified) 2- Yes (Experience Required) 3 - Yes (Fellowship Required) 4- Yes (Fellowship and Experience Required) |
| 35 | Orthopedic cases included | Categorical | 0 - Not specified 1 - Yes (Nothing Specified) 2- Yes (Experience Required) 3 - Yes (Fellowship Required) 4- Yes (Fellowship and Experience Required) |
| 36 | Urology cases included | Categorical | 0 - Not specified 1 - Yes (Nothing Specified) 2- Yes (Experience Required) 3 - Yes (Fellowship Required) 4- Yes (Fellowship and Experience Required) |
| 37 | "Bread and Butter" cases excluded | Categorical | 0- Case included 1 - Not specified 2 - Case excluded |
| 38 | Bariatrics cases excluded | Categorical | 0- Case included 1 - Not specified 2 - Case excluded |
| 39 | General cases excluded | Categorical | 0- Case included 1 - Not specified 2 - Case excluded |
| 40 | Cardiothoracic cases excluded | Categorical | 0- Case included 1 - Not specified 2 - Case excluded |
| 41 | Pulmonary/Critical Care cases excluded | Categorical | 0- Case included 1 - Not specified 2 - Case excluded |
| 42 | "Neurosurgery/Spine cases excluded | Categorical | 0- Case included 1 - Not specified 2 - Case excluded |
| 43 | Dental cases excluded | Categorical | 0- Case included 1 - Not specified 2 - Case excluded |
| 44 | Burns/Trauma cases excluded | Categorical | 0- Case included 1 - Not specified 2 - Case excluded |
| 45 | Transplantation cases excluded | Categorical | 0- Case included 1 - Not specified 2 - Case excluded |
| 46 | Pediatric cases excluded | Categorical | 0- Case included 1 - Not specified 2 - Case excluded |
| 47 | Podiatry cases excluded | Categorical | 0- Case included 1 - Not specified 2 - Case excluded |
| 48 | Pain management cases excluded | Categorical | 0- Case included 1 - Not specified 2 - Case excluded |
| 49 | Plastic surgery cases excluded | Categorical | 0- Case included 1 - Not specified 2 - Case excluded |
| 50 | Palliative Care/Geriatric cases excluded | Categorical | 0- Case included 1 - Not specified 2 - Case excluded |
| 51 | Regional anesthesia/Nerve blocks cases excluded | Categorical | 0- Case included 1 - Not specified 2 - Case excluded |
| 52 | Robotic surgery cases excluded | Categorical | 0- Case included 1 - Not specified 2 - Case excluded |
| 53 | Obstetrics/Gynecology cases excluded | Categorical | 0- Case included 1 - Not specified 2 - Case excluded |
| 54 | Otorhinolaryngology cases excluded | Categorical | 0- Case included 1 - Not specified 2 - Case excluded |
| 55 | Ophthalmology cases excluded | Categorical | 0- Case included 1 - Not specified 2 - Case excluded |
| 56 | Endoscopy/Gastrointestinal cases excluded | Categorical | 0- Case included 1 - Not specified 2 - Case excluded |
| 57 | Vascular cases excluded | Categorical | 0- Case included 1 - Not specified 2 - Case excluded |
| 58 | Orthopedic cases excluded | Categorical | 0- Case included 1 - Not specified 2 - Case excluded |
| 59 | Urology cases excluded | Categorical | 0- Case included 1 - Not specified 2 - Case excluded |
| 60 | CRNA Supervision Required at Facility | Categorical | x - Not specified 1 - Yes 2 - No |
| 61 | Starting Time of Shift | Continuous | Military Time |
| 62 | Finish Time of Shift | Continuous | Military Time |
| 63 | Total Number of Daily Working Hours | Continuous | Military Time |
| 64 | Type of Assignment | Categorical | x - Not specified 1 - Locum 2 - Locum to permanent |
| 65 | Starting Date of Assignment | Continuous | MM/DD/YYYY |
| 66 | Length of Assignment in Weeks | Continuous | Number |
| 67 | Board Certification Required | Categorical | x - Not specified 1 - Yes 2 - No |
| 68 | Board Eligibility Required | Categorical | x - Not specified 1 - Yes 2 - No |
| 69 | Fellowship Required | Categorical | x - Not specified 1 - Yes 2 - No |
| 70 | Prior Experience Required | Categorical | x - Not specified 1 - Yes 2 - No |
| 71 | ACLS Required | Categorical | x - Not specified 1 - Yes 2 - No |
| 72 | BLS Required | Categorical | x - Not specified 1 - Yes 2 – No |
| 73 | License Required | Categorical | x- Not specified 0- No 1- Yes 2 - Any State License is sufficient 3 - Temporary Privileges |
| 74 | Company | Categorical | Companies Coded and Withheld for Privacy |
| 75 | Daily Rate | Continuous | x - Not specified Number (given or calculated) |
| 76 | Not Applicable to Study | - | - |
| 77 | Travel Paid by Staffing Agency | Categorical | x - Not specified 1 - Yes 2 - No |
| 78 | Lodging Paid by Staffing Agency | Categorical | x - Not specified 1 - Yes 2 - No |
| 79 | Licensing Paid by Staffing Agency | Categorical | x - Not specified 1 - Yes 2 - No |
| 80 | Malpractice Paid by Staffing Agency | Categorical | x - Not specified 1 - Yes 2 - No |
| 81 | Expenses Paid by Staffing Agency | Categorical | x - Not specified 1 - Yes 2 - No |
| 82 | Number of Available Positions | Continuous | x - Not specified Number |
